# Exploring how children and adolescents talk about coping strategies relating to loneliness using reflexive thematic analysis: a qualitative study

**DOI:** 10.3389/fpsyt.2024.1462189

**Published:** 2024-11-15

**Authors:** Lauren Burke, Lily Verity, Laura Riddleston, Delia Fuhrmann, Pamela Qualter, Jennifer Y. F. Lau, Ola Demkowicz

**Affiliations:** ^1^ Manchester Institute of Education, The University of Manchester, Manchester, United Kingdom; ^2^ Youth Resilience Unit, Centre for Psychiatry and Mental Health, Queen Mary University of London, London, United Kingdom; ^3^ Psychology Department, King’s College London, London, United Kingdom

**Keywords:** children, adolescents, youth, loneliness, qualitative, coping strategies

## Abstract

**Introduction:**

The prevailing view is that loneliness predominantly affects older adults. However, awareness of high rates of loneliness among younger populations is growing, prompting a call for interventions. The current study aimed to listen to the voices of young people regarding how they cope with loneliness, gaining a better understanding of how to then develop tailored interventions.

**Methods:**

Thirteen Arts-based focus groups were conducted with 74 participants (8-18 years old), in London, Manchester, and South Yorkshire. Reflexive thematic analysis was utilised.

**Results:**

We developed six themes as follows: (1) “Determinants of the coping approach for loneliness”, (2) “Considerations to guide decision making”, (3) “Coping strategies to alleviate loneliness”, (4) “Social connection as a coping strategy for loneliness – considerations”, (5) “Being active in your own coping success”, and (6) “Worsening loneliness, coping strategies gone wrong”.

**Conclusions:**

Participants described a partially sequential process in choosing coping strategies for loneliness, including effective and maladaptive choices. Effective strategies were highlighted, reflecting developmental stages with the need for self-motivation. Participants noted challenges in engaging in coping due to skill deficits. These findings are crucial for developing interventions specific to this population.

## 1 Introduction

Loneliness is an unpleasant and distressing subjective experience. Distinct from the objective experience of social isolation, it arises from perceived deficiencies in an individual’s social relationships (1). There are adverse effects of loneliness on both the physical and mental well-being of children and adolescents (youth) (2, 3), and older populations (4, 5). There is still a widely held stereotype that loneliness exists primarily in older adults, despite evidence that younger age groups experience loneliness (6–8), and in some instances are the most lonely (9, 10). This implies that strategies aimed at addressing loneliness among youth potentially rely on drawing from research conducted with older populations because there are no known qualitative studies that directly explore coping strategies among youth. This has led to the implementation of interventions that may not fully align with the developmental stage that young people are in, including their needs and preferences. Such a deviation conflicts with established NICE (National Institute for Health and Care Excellence) guidelines and public health literature that acknowledges effective interventions should target the needs of specific groups, including whether they are age-appropriate (11–13). The aim of the current study was, therefore, to respond to this identified research gap and explore, using qualitative methods, how young people talk about coping strategies related to loneliness. By asking youth directly what helps relieve loneliness, we can establish a better understanding of how they cope with it, and more suitable interventions can be developed if appropriate.

The need to be connected to others is consistent across the lifespan (14). In addition, the negative emotions and cognitions experienced when this need is not met are comparable between people of different ages (15). However, there are different sources of loneliness at each life stage (15). For example, in the early stages of childhood, children place considerable significance on friendships with those in their peer groups, primarily centred around forming friends with individuals who are present at that time (16). As children move into adolescence, friendship quality becomes more important, with an increasing focus on the value of validation, understanding, self-disclosure, and empathy (15, 17). Throughout all of this time, affiliation within the peer group is also important, and increasingly so; children become increasingly concerned with belonging and being socially accepted by a peer group as they progress into adolescence (18, 19). An extreme form of peer group rejection linked to loneliness is recognised as victimisation (20). Hence, a lack of friends, low quality friendships, peer rejection, and victimisation are recognised predictors of loneliness as children transition into adolescence (21).

Changes in the sources of loneliness across development coincide with changes in how youth manage and cope with difficult experiences (22). Coping is defined as ‘conscious volitional efforts to regulate emotion, cognition, behaviour, physiology, and the environment in response to stressful events or circumstances’ (23). As the ability to cope is determined by the biological, cognitive, social, and emotional development of the individual, advancement in cognitive and emotional ability is likely to be associated with changes in coping strategies and mechanisms.

During childhood, we see transformations from a reliance on innate responses to aid coping in infancy (24) to sophisticated coping strategies, including cognitive reframing and problem-solving (25). By middle childhood and early adolescence, youth are typically increasingly matching appropriate coping strategies to situations (23). While there are challenges to defining coping and establishing a classification of coping strategies to provide a cohesive comprehension of the structure of coping among youth (23), the most commonly used classification systems are as follows: problem-focused (seeking information, taking action to change circumstances causing stress) versus emotion-focused coping (expressing emotions, seeking support and trying to avoid the source of stress (26); primary control (problem-solving, regulating emotional response) versus secondary control coping (adapting to the environment, including to seek acceptance (27, 28); and an engagement (responses orientated toward the source of stress, or the individual’s emotions e.g. problem-solving, emotional support) versus a disengagement ‘avoidance’ approach (orientated away from the stressor or the individual’s emotions e.g. withdrawal or denial (29, 30). Overall, research suggests that coping strategies will change developmentally, falling into different classifications, depending on individual differences and circumstances surrounding the stressor (22, 23).

To date, research has shown that therapeutic techniques and interventions can be effective treatments for mental health conditions in which loneliness is typically present (31). This includes anxiety and depression, where psychological interventions such as Cognitive Behavioural Therapy, Behavioural Activation, and Interpersonal Psychotherapy have proven to be effective for younger populations (32–37). However, it is important to acknowledge that loneliness was explored there as a secondary outcome, and whilst such research indicates potential effective coping strategies for loneliness, it overlooks what mechanisms within those interventions are working to alleviate loneliness and, in these instances, does not consider loneliness as a main concern.

Those diverse interventions identified as being successful at reducing loneliness among youth align with a range of the coping classifications stated above, indicating the potential necessity of a diverse approach to alleviating symptoms in this instance associated with depression and anxiety. Furthermore, this links to longstanding criticisms of such classifications as being overly broad (23). Despite shared emotional responses, developmental changes in the source of loneliness mean that certain techniques may not be beneficial when attempting to primarily reduce loneliness for different age groups. The implication is that different strategies may be required to implement successful coping strategies to overcome loneliness, and at different ages. The changing sources for loneliness during childhood to adolescence, and their differentiation from those during adulthood (15), emphasise the necessity for interventions that are tailored to specific age groups. Such interventions should be guided by the relevant theory, and incorporate coping strategies used and understood by the target age group to ensure their suitability for the targeted age group (11), as recommended by NICE and Medical Research Council (MRC) guidelines (12, 13).

To date, interventions for youth that aim to reduce loneliness have predominantly been shaped by quantitative research, in both their development and evaluation. Solely relying on such methods results in a fragmented and partial evidence base, hindering the creation of comprehensive intervention strategies. It has been highlighted that recent public health claims regarding loneliness have consequently initiated an influx of studies using limited survey data, that disregards the relevance of including an individual’s lived experience of loneliness (38). This hinders the broader body of research into loneliness by limiting the findings, and such research has advised the need for qualitative data to expand on and complement quantitative findings.

Qualitative research to date with youth in this area has generally focused on perceptions and experience of loneliness. One early study showed that youth experience loneliness relating to two key constructs revolving around friendship and their perception of being alone (39). Being alone was suggested to have both positive and negative associations influenced by factors such as setting and frequency, with friendship networks serving as a buffer against loneliness. Further qualitative research has shown that loneliness can lead to negative experiences like stress, shame, and exclusion (40, 41). Conversely, this evidence has highlighted that self-chosen ‘loneliness’ (spending time physically on your own) can have positive outcomes, including freedom, recovery, creativity and an opportunity to recharge (40). However, qualitative research that explores loneliness and coping strategies among youth remains limited. This means that we currently lack the depth and understanding that qualitative research provides in exploring the intricacies, meanings, and nuances of loneliness. A study conducted with 8-14 year olds is one of the only such contributions to this literature (42). Researchers identified coping strategies as split into two themes: those that aim to alleviate the negative emotional impact of loneliness (writing in a diary or engaging in an enjoyable activity), and those that aid social reconnection (involving connecting with peers, and adults helping facilitate this). The lack of qualitative research that captures the voices of younger individuals creates a substantial barrier to gaining an understanding of how they cope with experiences of loneliness, which is vital for progressing interventions that will be appropriate and effective for this population.

In the current qualitative study, we listen to the voices of youth to explore loneliness and coping strategies, which will inform future interventions work. We explore coping strategies used in relation to loneliness across the age groups to consider what strategies are suggested. The study will provide information about how youth cope with loneliness and what these strategies mean for intervention development.

## 2 Materials and methods

### 2.1 Design and context

We explore qualitative data produced from a study funded by the MRC (MR/X002381/1; 43) that included the development and validation of an age-sensitive scale of loneliness for young people ages 8-24 years: the Youth Loneliness Scale (YLS). Here, we use data from the early stages of the YLS multi-step project, where inductive item generation via arts-based focus groups was conducted to enable broad expression of youth around what it means to be lonely (43). Focus groups took place in London, Manchester, and South Yorkshire, and were arts-based, meaning participants reflected both verbally and through arts (in which both their narratives and creative material were recorded) on their experiences of loneliness. We note that we only include data from focus groups run with participants from the ages of 8-18 years old, and therefore in full-time education (i.e., excluding those who took part in separate groups to the ones in the current study for those aged up to 24 years).

The focus groups took place in social settings, with an understanding that loneliness, the topic of interest, is a social experience, and in alignment with the social constructionist epistemology. This enables recognition of how the personal experiences of the team shape our understanding of these discussions in line with this epistemological perspective. The contributions of the team are discussed in author contributions.

### 2.2 Participants

Data from 74 participants (77% female) ages 8-18 years, across 13 focus groups, were analysed (demographics are shown in **Table 1**). The inclusion criteria comprised individuals aged 8-18 of all genders, who had either experienced loneliness themselves or were aware of its meaning for others, were fluent in English, and had no neurological disorders (e.g., epilepsy, moderate to severe learning difficulties, or a history of serious head injury). The focus groups were conducted in 2023. Age categories were established according to educational progressions in the UK (44). Opting for more precise age ranges had the potential to optimise the process of prompting and facilitating conversations among participants. These age categories also allowed us to acquire a deeper understanding of the diverse manners in which loneliness was experienced across various stages of development. This allowed for the focus groups to select activities that were age-appropriate. For example, asking participants to express their answers through drawing or writing was a consistent task for all, regardless of age. However, activities such as passing a teddy around and creating a story were used only with younger participants. The selection of appropriate art activities was guided by the research team’s experience and feedback from the youth throughout the study. In addition, if the children were not engaging in a particular activity, we adapted our approach based on their responses. However, in some instances, groups differed in age range due to recruitment and time restrictions, but appropriateness of activities was still considered. If participants became distressed, a protocol was followed by the research team. The participant was taken out of the session and reminded that they did not need to continue to take part if they did not wish to. Multiple research team members were present so that the children remaining in the focus group were not left alone. However, the balance between researchers and participants was always considered. School staff and parents/carers (dependent on setting) were informed of the protocol enforcement and completed forms were reviewed with the YLS principal investigators to ensure proper handling. Participants were informed that any concerns would be shared if they arose. The overall YSL project was granted ethical approval to recruit 8-9 year olds in addition to their original plan, for focus groups to be conducted for the purposes of this current study. Participants were recruited to this stage of the YLS project via poster advertisement or their school. Posters had been designed for prospective participants, as well as parents or guardians. They were exhibited across university campuses (Queen Mary University, London and University of Manchester, Manchester; both UK), on the social media platform Twitter (or X), and in various local public venues (such as cafes, museums, etc.) and schools. Participants were invited to take part in focus groups in venues in summer 2023. Alternatively, schools known by the team to be research active were contacted and asked to share information by members of the research team to discuss potential involvement.

**Table 1:** Demographics of participants.

N	Age (years)	Number of females	Number of males	Number identifying as non-binary	Location of FG	Length of FG (min, seconds)
6	8-10	2	4	0	South Yorkshire	67.59
7	8-11	5	2	0	Manchester	123.48
7	8-11	3	4	0	London	112.59
4	11-12	1	3	0	Manchester	48.26
4	12-15	2	2	0	Manchester	82.03
5	12-15	3	2	0	London	104.23
6	12-13	3	3	0	Manchester	48.50
6	13-14	6	0	0	Manchester	42.55
6	14-15	6	0	0	Manchester	34.44
2	14-15	0	2	0	Manchester	24.33
2	16-18	1	1	0	Manchester	136.31
6	16-18	5	1	0	London	115.02
13	16-18	11	1	1	London	140.25

### 2.3 Data generation

Focus group schedules and arts activities were tailored for the different age groups. Interview schedules can be found in as part of the overall YLS study protocol **Supplementary Materials** available on the Open Science Framework (OSF) from https://osf.io/4qvnh/ (43). Interview schedules are split as they were adapted based on location (school vs. summer venues). These questions were designed to probe (a) behaviours, emotions, and cognitions associated with loneliness, and (b) relevant and variable dimensions of these experiences (frequency, intensity, duration, emotional impact, coping strategies), and were followed with prompts to explore responses more in-depth where necessary. Authors were involved in designing the questions to involve coping strategies to allow for the current study to be undertaken. Facilitators with experience of conducting arts-based workshops ran the focus groups in university-approved venues over the summer. Researchers observed those sessions, facilitating further focus groups that took place in schools. Focus group timings varied based on the location (24 minutes 33 seconds – 140 minutes 25 seconds); school-based groups were shorter sessions (70 minutes or less). Participants who took part in the study outside of school over summer received a £40 voucher as a thank you; participants in schools did not receive an individual voucher, but the school received a £100 voucher overall for each group of pupils who completed focus groups.

### 2.4 Ethical consideration

The overall YLS project received ethical approval from Queen Mary University (reference: QMERC23.065). Participants over 16 years of age or parent/guardian of the child in the focus group stage of this project were invited to register their interest online via a link. Written consent was obtained for all participants prior to the focus groups; where participants were under 16 years written parental/guardian consent was obtained, and assent was obtained from the participants.

### 2.5 Data analysis

Data were analysed using Braun and Clarke’s six-stage reflexive thematic analysis (RTA) (45), which is concerned with patterns, themes, and meanings within data. Additionally, it places significant emphasis on the component of reflexivity, facilitating researchers to engage in critical reflection on their values and assumptions during the research process (46). The authors involved in the analysis all have prior experience of utilising this methodology. The current study follows a social constructionist perspective, as outlined above, to accommodate the social experiences surrounding the context of loneliness occurring as well as the environments in which the participants took place in these focus groups. This research is relatively exploratory, as there is a lack of qualitative research exploring coping strategies for loneliness in this age group. An exploratory approach aligns well with RTA which functions exclusively in a qualitative paradigm, and in this analysis unconcerned with prior theme development or reliability. This allows the following analysis to be driven by the data, whilst recognising our roles as researchers. The current study will be taking an inductive approach to complement the exploratory aims and approach of the current study. This allows for the development of patterns based on the data collected rather than being guided by pre-determined assumptions or research. RTA was implemented here with a ‘latent’ angle and involves researchers looking beyond expressed statements of participants and looking for underlying themes, patterns and meaning that may not be initially evident in explicit statements from participants (45). Instead, we explored implicit aspects and hidden points of participants’ expressed experiences to allow for a deeper understanding of coping strategies for loneliness.

Analysis via the six-step RTA approach was organised using NVivo Version 12. Authors LB, LV, and LR familiarised themselves with the data, in LB’s case with a particular focus on coping; LV and LR familiarised themselves for the purposes of undertaking analysis for the overall YSL project. This prompted the decision to include full transcripts in the analysis, rather than focusing only on focus group questions that addressed coping, as it was evident that this topic was present in responses to other questions, and was not limited to those questions explicitly on coping. LB generated initial codes, in line with the research question. Any artwork created during the focus groups was reviewed when LB generated the codes and complemented the transcript data. For example, participants drew portraits of loneliness and discussed those. LB ensured this artwork was accessible when coding this section of the transcript. Authors LV and OD contributed to generating initial codes, as part of reflexive discussions. LB maintained a reflection log, which was shared in meetings.LB noted areas of uncertainty regarding the coding of specific sections of the transcript, and these were resolved in discussions with OD and LV. This process was devised in line with the key concepts of RTA, in which ‘quality is not dependent on multiple coders’ or concerned with reliability (47). A total of 362 codes were generated across the dataset. The third step collectively explored how the codes would be organised into themes. Author LB initially considered this step, bringing thoughts to the whole research team for a collaborative reflexive discussion. Finally, LB reviewed and refined those themes through such discussions and confirmed with the research team. Codes were checked against the overall theme name, ensuring the suitability of codes according to the research teams thoughts and research aims. Following those meetings with all team members, Author LB, then, produced a coherent report that conveyed the identified themes and their significance.

## 3 Findings

We developed six main themes: (1) “Determinants of the coping approach for loneliness”, (2) “Considerations to guide decision making”, (3) “Coping strategies to alleviate loneliness”, (4) “Social connection as a coping strategy for loneliness – considerations”, (5) “Being active in your own coping success”, and (6) “Worsening loneliness, coping strategies gone wrong”. Ten sub-themes were identified, nested within specific main themes.

**Figure 1:**
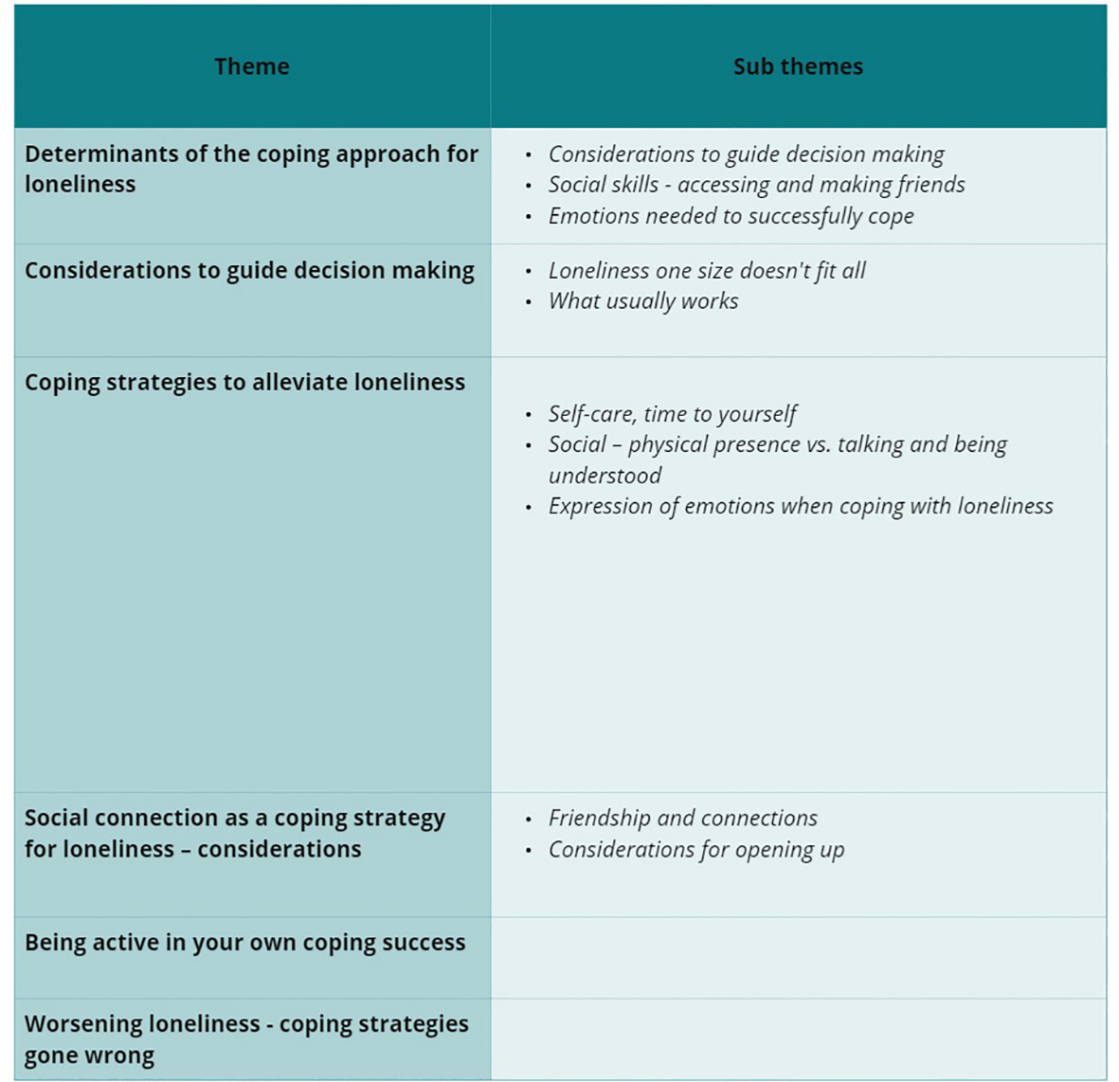
Outline of themes and sub-themes.

**Figure 2:**
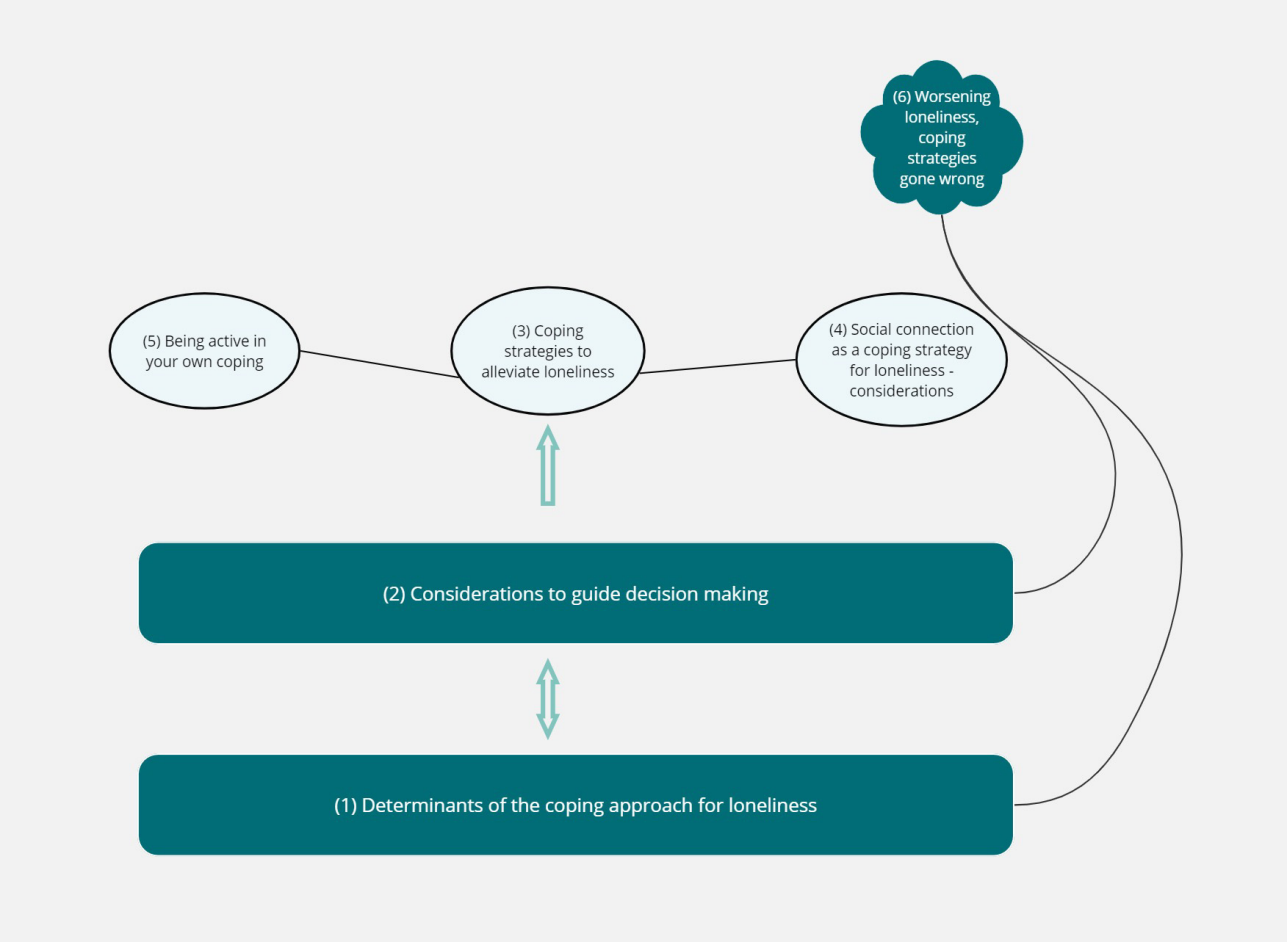
Illustration showing connections between themes.

An outline of the themes and sub-themes can be found in **Figure 1**. **Figure 2** illustrates how the themes are connected. This figure has been simplified for clarity, showing only the main links and relationships. Themes are laid out to recognise the prevalence of themes 1 and 2 throughout and how they are interlinked with the other themes. Participants navigate these determinants and considerations, which guide them toward either theme 3 or 6. This highlights that themes are somewhat sequential. The arrows illustrate how participants described the flow between themes, leading to decisions about coping strategies. Arrows indicate the process starts with themes 1 and 2, with a double-headed arrow showing their interconnection, as participants moved back and forth between them before proceeding to themes 3 or 6. Themes 3, 4 and 5 are linked, representing components that make the selected coping strategy successful. Theme 6 is described as arising from themes 1 and 2. However, participants noted that the way theme 6 arises differs from the development of a successful coping strategy (theme 3) to alleviate loneliness. The size and shape of the illustration emphasise the description rather than the importance of themes.

During analysis, differences in participants’ thoughts across different age groups emerged in discussions about specific topics. Where that occurred, comments were made to highlight different thematic findings across age groups to aid the transferability of the qualitative findings. When providing quotations, the age of the participant is noted; that is offered as context rather than intending to indicate that the theme heavily reflects one age group as opposed to all participants, where that is the case, it is specifically noted.

### 3.1 Expression of loneliness in the focus groups

For readers to engage in the paper, we briefly offer context as to how participants spoke about loneliness. Essentially, participants spoke about loneliness negatively, relating it to undesirable emotions, and discussed how there were differences in the source of loneliness. An expanded reflection of this can be found in the **Supplementary File 1** in **Supplementary Table 1**.

### 3.2 Theme 1: Determinants of the coping approach for loneliness

#### 3.2.1 Identifying loneliness

Participants spoke about the role of decision-making before engaging in a coping strategy. They expressed how loneliness needs to be recognised, allowing time to reflect and choose a coping strategy:

“*You just need to like have some time by yourself and then come back to it*” [8-11 years].

Older participants (16-18 years) acknowledged how recognition of loneliness can be difficult, especially when you are younger:

“*I didn’t recognise it as loneliness” [but rather] “sadness and anger*” [16-18 years].

#### 3.2.2 Social skills – accessing and making friends

Participants talked about not always having the required skills to cope with loneliness. They reported that when lonely, it is difficult to initiate a coping strategy if you do not have the confidence, skills, or experience to do so. Socialising was specifically mentioned as something that participants knew had the potential to be an effective coping strategy, but is difficult to participate in if they do not have others available to socialise with, or experience in knowing how to socialise:

“*for some people, they don’t really know how to be social. Like, they won’t I guess they won’t be around that many people they have like a small close family, no family friends visit them often, only the occasional one, and they just don’t know how to be social with other people*” [12-15 years].

This discussion was directly expressed by participants from the years of 12-15. Participants ages 8-11 years expressed a want for certain things that they thought had the potential to be a coping strategy, but they knew they might not have access to. For example, a quote from a task in which participants were asked to tell a story regarding how to alleviate loneliness:

“*really wished he had some friends to talk to*” [8-11 years].

#### 3.2.3 Emotions needed to successfully cope

Participants described how a strategy needed to make you feel successful in alleviating loneliness. There were conversations based on how such strategies needed to be mood-lifting and increase happy emotions to alleviate loneliness. When describing using prayer as a coping strategy, a participant stated and then confirmed this would help with loneliness:

“*you could be lonely. But then erm if, if your like to praying God could make you feel happy*” [8-11 years].

Participants also brought up coping strategies needing to elicit feelings of calmness and make someone feel comfortable and understood to successfully alleviate loneliness.

### 3.3 Theme 2: Considerations to guide decision making

#### 3.3.1 Loneliness one size doesn’t fit all

Participants talked about how there were different types of loneliness. They noted that it differed by circumstance, that different feelings surrounded loneliness, and that different needs should to be considered when selecting an effective coping strategy:

“*I think that loneliness is a different experience every single person because they’ve experienced it in a different way, compared to another person who’s experienced it, and now two people who’ve experienced loneliness, it’s not the same for either one, even if they discussed it*” [12-15 years].

#### 3.3.2 What usually works

Reflection on what has worked previously for coping with loneliness was talked about by the oldest group of participants aged 16-18 years. They discussed how you could consider times you had previously felt lonely, and what worked to alleviate this to work out which coping strategies may be effective to reduce feelings of loneliness when or if they reoccur.

“*seeing things that you used to do during that time to cope*” [16-18 years**].**

There was also an acknowledgment from this age group that loneliness can be more of a long-term experience for some individuals. They recognised that when implementing previously successful coping strategies, the surrounding cause of loneliness at that time needs to be considered to see if it is as effective at present. Loneliness was described as:

“*something that you know, stays with you until you don’t like, until you address the root of the issue*” [16-18 years].

### 3.4 Theme 3: Coping strategies to alleviate loneliness

**Figure 3:**
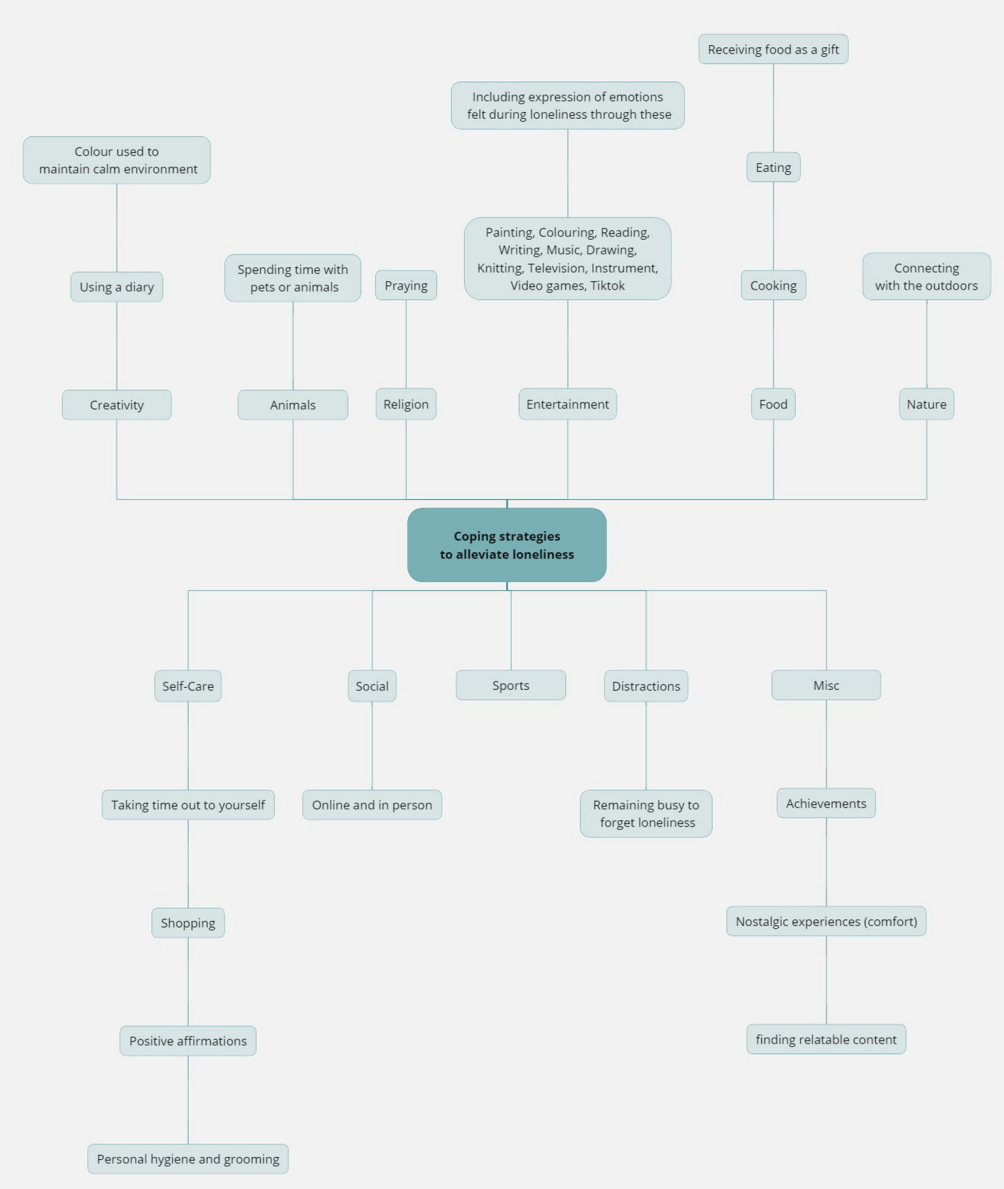
Suggested coping strategies by participants.

Participants spoke about coping strategies that could be utilised to alleviate feelings of loneliness. As part of analysis, suggested coping strategies were coded and later organised into categories based on the data, rather than having any pre-determined categories. These 11 categories are illustrated in **Figure 3**. The following three sub-themes explore and provide details for this overall theme.

#### 3.4.1 Self-care, time to yourself

Self-care, in particular taking time for yourself to think and reflect, was a strategy suggested by all age groups. However, younger participants from 8-11 years expressed how this may provide an instant relief to a form of short-term loneliness:

“*So it’s showing that you can just take a breather, or then you’ll be fine to go back and play again*” [8-11 years].

In contrast, older groups from 13-14 years spoke about how this was more of a reflective space to figure out your own needs:

“*just like being alone for a little bit ‘cause it might get a bit overwhelming, like having loads of friends always around you and always talking to you. It might feel good to like, be on your own for a little bit and, like, do what you want*” [13-14 years].

All participants said that spending time alone can serve as a coping strategy for loneliness, allowing time to reflect, or undertaken activities alone which you enjoy. Additionally, others emphasised the distinction between solitude and experiencing loneliness, noting that enjoying time alone does not necessarily include negative feelings, unlike loneliness. This process was expressed across all age groups, however, younger groups (8-11 years) seemed to describe spending physical time alone and being happy as loneliness too:

“*When you when you’re alone. Like you can be quite content, whereas if you like facing loneliness, it’s more like a feeling of isolation*” [14-15 years].

“*Because sometimes you want to be lonely. As in like you might wanna have time to yourself which I definitely do sometimes*” [8-11 years].

#### 3.4.2 Social – physical presence vs. talking and being understood

Participants suggested socialising with others as a coping strategy. Spending time with friends and family was suggested by all ages, with participants expressing how just being in the presence of others (especially friends and family), including physical contact and affection specifically can help.

“*I share a room with my three younger brothers … So that’s why I don’t feel lonely. I just have a subconscious feeling knowing that my brothers are just there*” [12-15 years].

There was a focus on being included to alleviate in loneliness, particularly through socialising via playing games. Participants in older groups (12-15 years) described how they would need to be engaging in conversations with their friends to be able to benefit from socialisation as a coping strategy:

“*I think everybody feels a bit less lonely when they’re with like family or friends. Or people they just haven’t seen in a long time. That they have a relationship with*” [12-15 years].

Furthermore, older groups expressed the need for “*your parents and family to understand you*” [16-18 years], to potentially benefit when talking and socialising with them as a strategy.

#### 3.4.3 Expression of emotions when coping with loneliness

Those 11 years and older discussed loneliness as associated with a range of emotions, including sadness, frustration, and anger. Participants said that the emotional experience can differ for everyone and depends on the trigger for loneliness. Emotional expression was touched upon as being a coping strategy for some individuals. Others expressed the importance of considering the range of emotions loneliness is associated with when choosing an appropriate coping strategy:

“*I don’t really feel loneliness as a sort of solo emotion. It’s more just, I will feel lonely … mixed with sadness and other things like that. So it’s never really just loneliness*” [16-18 years].

Anger was picked up on by some of participants, and how loneliness can feel frustrating:

“*Anger, because it feels like everyone’s moving on without you. And you know you can be better than this, but you’re stuck in that space of inactivity*” [14-15 years].

Participants also expressed an appreciation for the emotions; the discomfort of experiencing negative emotions acted as motivation to overcome loneliness:

“*I’m so grateful that I can feel sadness so then when I feel happy. I have those extremes of emotion and it’s like a spectrum. So sometimes when I feel lonely, I’m actually like, it spurs you to them, then want to feel unlonely*” [16-18 years].

### 3.5 Theme 4: Social connection as a coping strategy for loneliness – considerations

#### 3.5.1 Friendship and connections

Participants described how connecting, and socialising with friends can be a coping strategy (as referred to in theme 3). This ranged from simply spending time with them to undertake an activity together, to support to discuss their feelings or being in their presence. However, participants also expressed how there are some considerations around these connections to ensure they worked as a strategy to reduce feelings of loneliness. Participants discussed the need to be selective in choice of interaction, and the specific qualities friends must possess to serve as a coping strategy. These qualities include the ability to compromise, shared interests, trustworthiness, reliability, active engagement, closeness, and authenticity.

Younger groups (8-11 years) spoke about how friendships as a coping strategy include having other children to play with at the time, and negotiating to not be left out, particularly during games.

“*if you don’t have any friends then if you’re lonely then you won’t have anybody to talk to or you won’t have anybody to play with*” [8-11 years].

Participants depicted loneliness by drawing children excluded from play. They discussed rejoining the game as a solution to alleviate loneliness.

In older focus groups (above 11 years), there was a shift to not only spending time with your friends but also using them as a support and expressing your feelings to aid coping. In addition, there was an appreciation of how these friendships require work in these older groups. There were discussions around how you can’t expect your friends to do everything for you, and there is a mutual effort in friendships which makes them successful to be able to be utiilised as a coping strategy.

“*You can have friends, but you need to work with them to become better friends. You can’t. You can’t expect them to do everything for you and expect them to be still friends with you*” [14-15 years].

These older participants also talked about how to be able to use friends as a coping strategy in this way, they need to be non-judgmental and offer reassurance that you are confiding in someone who wants to listen.

However, some participants expressed that confiding in a stranger rather than a friend, or someone they know might be an easier coping strategy for loneliness, to facilitate a discussion around feelings.

“*I think it can be easier to open up to a stranger because you don’t know them and they don’t know anything about you*” [14-15 years].

Additionally, there was further awareness from all participants of how friendships were highlighted as something that can help but also be the cause of loneliness, and potentially make you feel worse when frictions arise. Other examples raised included the use of online spaces to maintain friendships, and forcing friendships where you have no common interests.

#### 3.5.2 Considerations for opening up

Participants discussed the qualities they seek in confidants when expressing their feelings, which was proposed as a coping strategy for loneliness. Participants expressed a need to find trustworthy individuals to confide in and to feel safe, comfortable, and supported in their interactions, preferring those who were also relatable.

“*someone who knows what it’s like*” [12-13 years].

There was a sense that, especially in older participants (above 11 years), these qualities were in the back of participant’s minds when going to family members or other adults for potential support or with new acquaintances who were not yet friends. There was talk about finding your people, and almost how these qualities need to be apparent from the onset in someone and maintained to then form a friendship.

### 3.6 Theme 5: Being active in your own coping success

Participants explored the view that the ability to employ successful coping strategies and mitigate feelings of loneliness had to be primarily self-motivated. Participants acknowledged the effectiveness of being able to identify your wants and needs when navigating decisions around how to successfully cope. It was also suggested by some that having a strong sense of self-worth, prevented someone from feeling lonely.

For others, effective coping with loneliness was difficult as it could be blocked by a negative view of yourself, and social interactions that arose when feeling lonely.

“*as much as you can tell someone you can go up to them and be like oh hi we think you’re nice sometimes it’s like you’re not always going to believe it*” [16- 18 years].

The need to put yourself out there, particularly when utilising social connection as a coping strategy was recognised.

“*Well, I think it depends on if you are like have the right mentality to try and make it better, but at the same time it’s it’s pretty hard to like change it. I think it just depends on like your mentality and how you’re feeling at that point of time*” [14-15 years].

It was also spoken about by some participants that this process, of almost self-discovery is important for learning what coping mechanisms work in the future, and how there is a need to be consistent in the ones that work for you. There was a sense that commitment and work was required from the individual to be able to overcome loneliness.

### 3.7 Theme 6: Worsening loneliness, coping strategies gone wrong

Participants noted that there are occasions when you feel lonely and take part in coping strategies that can make these feelings worse. Some older participants (16-18 years) recognised how you can be aware that you are doing this, and it can become self-destructive. These participants also expressed how it was hard to engage in the coping strategies they knew would make them feel better due to how bad loneliness makes you feel in the moment, highlighting the careful consideration for coping strategies.

Participants overall talked about how acting a certain way to fit in with friends was potentially a coping strategy for loneliness, however this likely leads to feeling worse:

“*If you think you’re lonely sometimes trying to fit in is worse because then it’s like you’re forcing yourself into these friendships where if you find someone that’s just like balances you out someone with, like, you’re happy around then. I think that would definitely help with friendships and loneliness*” [13-14 years].

This linked to conversations around how it was sometimes easier for some participants to engage in masking loneliness as a coping strategy. There were conversations about how you can be judged for taking part in certain coping strategies. Participants expressed this as “*Putting on an act*” [12-13 years], and pretending that they did not feel lonely, or hiding from their feelings:

Some participants noted how doing the above, can make them withdraw and ultimately lead to feeling worse. Some participants expressed a fear of taking part in potential coping strategies that may not work for them and fail. Others talked about how the idea of raising awareness, in this case of loneliness can be triggering and potentially lead to the worsening of loneliness.

“*if you have, like, an assembly, what about loneliness and how you need to talk to about it, and it might make you feel like pressured into, like needing to do it when you think I can’t do that because otherwise I’ll just panic and react*” [12-13 years].

Moreover, during a task where participants had to describe a story about how to alleviate loneliness, many stories from participants ages 8-11 years depicted scenarios where individuals experienced loneliness due to being left out, followed by a breakdown in friendship, exacerbating their feelings of loneliness. Older groups (16-18 years) specifically reflected on how it may become harder to engage in coping strategies which have the potential to alleviate loneliness if you have felt this way for a long time. They also expressed (14-18 years) feelings of exhaustion, helplessness and not wanting to reach out for help can be barriers to engagement in such strategies.

## 4 Discussion

To the best of our knowledge, this is the first study to explicitly explore coping strategies used by youth (8-18 years of age) in the context of loneliness. Our analysis of data from focus groups described a decision-making process for loneliness, where some aspects occurred sequentially, reflecting the individuality of loneliness experiences. Youth in the current study explained limitations in skills and resources hindering effective coping strategies, but also noted valuable coping strategies that included socialising with friends who met desired qualities, such as trustworthiness. Participants highlighted personal agency and self-worth in successful coping, whilst explaining how some strategies may exacerbate loneliness, and how an individual may still take part in such strategies despite knowing that. Findings show how coping strategies are carefully chosen by individuals to meet their needs and circumstances, to successfully alleviate feelings of loneliness.

Previous research has reported on strategies for dealing with loneliness among youth (8- 15 years) (39, 42), and adult populations (48), but the current study reports on the decision-making process youth say they use to cope with loneliness. The process described by youth in the current study can be understood within the framework proposed by Lazarus & Folkman (26), which outlines how stress responses are influenced by individual appraisals. Within that model, when presented with stressors, individuals evaluate their significance and their resources to manage them, which impact the decision-making surrounding the chosen coping strategy (49). Additionally, this involves a sequential element where certain decisions must be made to select and undertake a coping strategy, whether it proves effective or maladaptive. Recognising this pathway is crucial for future studies to develop effective loneliness interventions with this theoretical framework in mind. Exploring the potential sequential elements could guide interventions targeting specific stages of the decision-making process ensuring youth select effective coping strategies.

Participants noted a lack of skills or the unavailability of resources (such as friends) as barriers to engaging in a coping strategy that they believed could reduce loneliness. This echoes previous research (42). Combined, those findings suggest that interventions focused on social and emotional learning (SEL) are likely to be effective if they build youth’s confidence to manage the emotions that characterise loneliness and choose effective coping strategies. SEL interventions aim to develop social and emotional skills (e.g. self and social awareness, self-management, relationship skills and decision-making) within learning environments such as educational settings (50), and a review of interventions showed that those with components of SEL were particularly beneficial for reducing loneliness among youth (51). Additionally, an RCT has shown that a widely used SEL intervention has significant positive, maintained, effects on youth’s loneliness (52). We show that the voices of young people support that work. We also expand previous work finding that older youth directly mentioned a lack of skills as a problem, while younger youth indirectly described what they needed to do to cope. This highlights the importance of ensuring interventions are age-appropriate, in line with NICE guidelines and public health literature (11, 12).

Participants discussed various ways to lessen the emotional impact of loneliness, such as seeking social support, expressing themselves creatively, practicing self-care, engaging in cooking/eating, finding distractions, and turning to religion (praying). Some of those strategies, according to Lazarus & Folkman (26), serve as both emotional and problem-focused coping, suggesting that youth cope with loneliness in different ways based on context, time, and development. For example, spending time alone can involve either reflecting on problems and solutions (problem-focused) or seeking relaxation (emotion-focused). Similarly, socialising can entail seeking emotional support through the expression and validation of feelings (emotion-focused) or seeking solutions and building networks (problem-focused).

Research in the field of coping tends to favour problem-focused coping, as opposed to emotion-focused coping, as being more effective at mitigating the effects of stressors (53, 54). A literature review (48) on the association between loneliness and coping strategies has shown that. However, the review focuses on those over 18 years, emphasising the need for further research in younger populations where studies are scarce. Given that problem-focused coping is developed throughout childhood and adolescence, becoming more sophisticated based on life experience (26), it is no surprise that younger participants tended to describe more emotion-focused coping in the current study, describing taking breaks to regulate their emotions and relying on socialising with those in their surroundings to engage in activities like games to alleviate loneliness (short-term distraction). Conversely, older participants showed a greater tendency toward problem-focused coping, utilising reflective spaces to make decisions and social relationships to express their emotions (emotion-focused), whilst also seeking support and advice (problem-solving). These findings have important implications, highlighting that coping strategies considered effective for adults cannot necessarily be generalised to younger populations. They also underscore the potential for social and emotional learning (SEL) interventions to teach coping strategies. While such interventions should focus on teaching coping strategies that have shown to be effective, there needs to be a consideration for emphasising age-appropriate coping skills that align with their current abilities, rather than introducing strategies that exceed their developmental capabilities.

Participants also described engaging in maladaptive coping strategies. Participants discussed how people may not be aware that they are doing this at times, and, therefore, worsening their loneliness, perhaps because of the shame surrounding loneliness. Examples of these strategies include pretending to not feel lonely, or forcing friendships where you did not fit in. Previous research outlined similar findings where participants (12-18 years) expressed not showing show their full emotions at school, and feeling their friends did not know the ‘real’ them as a result (41). Such findings contribute essential knowledge about identifying potential signs that young people are engaging in coping strategies that could exacerbate their feelings of loneliness. This is crucial for developing interventions because it enhances understanding of those who may appear to be functioning well but are not. Moreover, it highlights that support can be provided through interventions, but also potentially by educators and guardians who can watch for these signs and improve their ability to communicate with individuals at risk of using such strategies.

Relating to those findings (41), participants in the current study expressed that the success of a coping strategy relied on self-motivation – success is about being able to identify your wants and needs related to your experience of loneliness. This was especially evident when participants discussed using social connection as a coping strategy, emphasising the importance of strong self-worth to initiate social interaction (as coping is an individual’s own responsibility). Conversely, negative self-worth was noted as hindering accepting positive overtures for social interactions: individuals may struggle to believe that such interactions are possible, being driven by a belief that others are distrusting and that they are not worthy of social relationships (15). Such findings, again, relate to those found in the studies above (40–42), and are further important considerations when adding to the knowledge of how young people cope with loneliness as well as for intervention development.

Socialising was a particularly prevalent narrative running through the participants’ descriptions of coping strategies in the current study, in line with previous findings from qualitative research amongst 8-18 year olds (41, 42). In their narratives, all participants described socialising as an emotional-focused coping strategy. However, older participants described how it was incorporated into their coping inventory as both an emotion-focused and problem-focused strategy, in line with expectations (26). Participants additionally described qualities individuals needed to maintain existing friendships or establish new ones, and, therefore, be part of a coping strategy involving socialising. Such qualities include the ability to compromise, shared interests, trustworthiness, reliability, active engagement, closeness, and authenticity. Older participants specifically emphasised essential traits needed for socialising as a problem-focused coping strategy, including the addition of needing non-judgemental and reassuring friends. This aligns with existing literature discussing the various sources of loneliness, whereby youth become more concerned about the quality of friendships as they develop (15, 17). Additionally, youth become more concerned about being accepted by a peer group as they age (18, 19). This relates to the above point regarding utilising fitting in and ignoring loneliness as a coping strategy, suggesting that maladaptive coping strategies are linked to a child’s developmental needs, specifically regarding friendship. Participants highlighted that while forming friendships can be an effective way to cope with loneliness, there is a delicate balance, as these relationships can sometimes also contribute to feelings of loneliness. This suggests potential important implications for intervention development and further emphasises the need for research to explore coping strategies for loneliness specifically within the population of a targeted intervention. For example, the connections between developmental stages and coping strategies highlight the significance of avoiding generalisations from the existing literature on older populations (where most loneliness research is conducted) to younger individuals.

### 4.1 Strengths and limitations of the study

Listening to the voices of children and adolescents from different age groups, gender groups, and areas in England are key strengths of the current study. Research shows that using arts-based approaches in focus groups helps participants reflect on and describe their experiences, specifically improving confidence and allowing youth to disclose problems that previously may have been hidden (55, 56). In the current study, providing different reflection outlets engaged children, and reminding them to use arts resources when quieter proved useful. Analytically, using RTA, the current study provides valuable insight into understanding coping strategies concerning loneliness by highlighting the perspectives of youth, a group persistently underrepresented within loneliness research. Recognising and understanding the nuances of coping strategies within this population can help us develop and adapt interventions specifically tailored to address loneliness within this population. This approach contrasts with relying on research from other populations, or interventions which are effective for existing mental health problems where loneliness is typically present.

However, there are also limitations to the current study. The participant group was predominantly female, which introduces a potential limitation to the generalisability and representativeness of the findings. Given established evidence of differences in how boys and girls tend to approach friendships and help-seeking behaviours (57, 58), the findings may not fully capture the difference in perspectives between genders. This gender imbalance is particularly relevant in the context of loneliness research, where much of the existing literature has focused on gender differences in the experience of loneliness but has not explored the differences in coping strategies, nor how such differences in coping strategies may occur (e.g., through socialisation). Future research should aim for a more balanced gender distribution to investigate these potential differences and enhance the applicability of the findings across genders. In addition, the inclusion criteria did not require participants to feel lonely at present or ever to take part. Questions were framed to allow participants to speak about their own experiences of loneliness, or from a third-person perspective (e.g., through asking what advice they would give friends, or individuals of a similar age), which provided a safe space for participants to share their thoughts. Future research should consider targeting lonely individuals to conduct qualitative research to build on the findings of the current study. Furthermore, the study’s reliance on arts-based activities in focus groups may have led to varying levels of participant engagement, potentially affecting the depth of data collected. However, facilitators and researchers offered alternative activities and tailored sessions to ensure comfort, and participants were informed about the arts focus during recruitment. Therefore, for future research utilising such methods, it is important to monitor and tailor accordingly to ensure engagement with the arts.

Although the authors collaboratively developed the questions on coping strategies to be included, this was not the focus of the larger study. Consequently, a limitation is that there may be a lack of depth to the discussion surrounding coping strategies. Another limitation is the varied settings for focus groups, as some took place in schools, while others were invited to participate over the summer and may not have known the other participants. This could affect responses since questions did not consider whether coping strategies were context specific. Some participants noted this, but we couldn’t presume that members of school-based groups were exclusively discussing coping strategies related to school.

Future research should consider the inclusion of more questions relating to coping strategies, with reference to settings. This would provide further depth to some of the findings. Although, given the current study was one of the first to specifically explore coping strategies within this population this design was beneficial. The exploratory approach not only offers valuable findings but provides insights for future research methods which is also valuable for further developing the field. Additionally, future research should also examine maladaptive coping strategies in youth loneliness because understanding these strategies is crucial for developing effective interventions. The findings of the current study highlight the importance of identifying why and how maladaptive strategies are chosen because they can worsen loneliness, potentially leading to long-term mental health issues. This comprehensive approach will enhance the effectiveness of interventions by addressing both effective and ineffective coping mechanisms.

## 5 Conclusions

Although the existence of loneliness within children and adolescents, as opposed to other populations, is gaining more widespread attention, particularly within research, there are still critical gaps including qualitative studies that listen and amplify the voices of this population regarding their experiences and coping strategies relating to loneliness. Participants in the current study described a process that enabled coping with loneliness; it is partially sequential process to deciding upon a coping strategy in youth. However, this process does not necessarily always lead to selecting an effective coping strategy. In some cases, maladaptive coping strategies can be selected. Participants discussed effective coping strategies tailored to their developmental stage, emphasising the importance of self-motivation in their implementation. They also noted how lacking certain skills can make engaging in loneliness-alleviating strategies, like socialising, difficult. Socialising was described often, and as serving various coping roles, reflecting developmental differences and highlighting how skill deficits might hinder effective coping strategies. Such findings are crucial in increasing youth voices and therefore contributing to effective intervention development, as research generalised from other populations cannot be relied upon to accomplish this. The current study therefore offers a novel and significant contribution to improving knowledge and understanding of how younger populations cope with experiencing loneliness.

## Data Availability

The original contributions presented in the study are included in the article/**Supplementary Material**, further inquiries can be directed to the corresponding author/s.
