# Supplementary material for: Exploring how children and adolescents talk about coping strategies relating to loneliness using reflexive thematic analysis: a qualitative study
Source: Front Psychiatry. 2024 Nov 15;15:1462189. doi: 10.3389/fpsyt.2024.1462189 (PMC11604979; doi:10.3389/fpsyt.2024.1462189)
Supplement: Supplementary file 1 [file Table1.docx]

**Supplementary Material File 1.** **Expression of loneliness in the focus groups**

The following reflection was not part of the formal analysis on the current paper, although authors were familiar with the transcripts. Below, LB provides an overview of expressions regarding loneliness in these participants, which was reviewed and agreed upon by the research team.

When asked what loneliness meant for them, participants expressed negative emotions which often occurred with isolation, younger participants referred to sadness, anger and getting upset, whilst drawing dark weather scenes with storms that blocked any positive/happy emotions (illustrated as sunshine). Older groups utilised more sophisticated language such as feeling drained, exhausted, frustrated, and overwhelmed. These groups also spoke about loneliness feeling like there is a void, weight, or emptiness within you. Across all age groups participants spoke about feelings of loneliness existing alongside mental health symptoms such as anxiety or depression, with an awareness of a reciprocal relationship between loneliness, and feelings of anxiety or depression. Stigma and shame surrounding loneliness was expressed by the groups. There was discussion around whether feeling lonely and being alone meant the same thing. All groups shared their thoughts on the distinction between the two concepts. They addressed how you can feel lonely in the presence of others, whereas being physically alone does not necessarily always mean you feel lonely. In certain situations, participants spoke about how being alone may be essential for their well-being and feeling good. The latter was not always considered to be loneliness; older groups explicitly understood that distinction whereas younger participants had to be probed for further understanding and would refer to ‘loneliness not always being a bad thing’, when describing being ‘alone’:

“*You can be in a room with crowd of people and you can still feel so alone*” **[16-18 years].**

“*So like when you're alone, you could like sometimes want to be alone or still be enjoying myself. Like you could have just wanted to be alone for a certain moment of time*” **[12-13 years].**

“*Sometimes it's good for you to be lonely because say you're like at a party and then like there's loads of people there like you all here shouting and like you just like ahhh, you just need to like have some time by yourself and then come back to it*” **[8-10 years].**

Participants expressed difficulties in friendships to be a source of loneliness. Accounts differed across age groups, with younger participants reflecting on being left out in social situations (games). As they got older loneliness was expressed as not fitting in, and the oldest groups spoke about the quality of friendships. With this came discussions around how long loneliness lasted, younger participants would talk about loneliness being shorter, whereas older groups expressed an appreciation that loneliness can last longer and potentially reoccur if not dealt with.

“*It depends how bad it is but say if it is really bad probably about, like 10 minutes, or if it's not that bad at all, like mine, that it could just be five*” **[8-10 years].**
